# Modal Superposition-Induced Novel Directional Responses in a Low-Damping Biomimetic Microphone for Sound Source Localization

**DOI:** 10.3390/s26113613

**Published:** 2026-06-05

**Authors:** Dipeng Ren, Xiaonan Yang, Zhi-Mei Qi

**Affiliations:** 1 The School of Electrical and Information Engineering, Zhengzhou University, Zhengzhou 450001, China; raintemple@zzu.edu.cn; 2 The State Key Laboratory of Transducer Technology, Aerospace Information Research Institute, Chinese Academy of Sciences, Beijing 100190, China; 3 The School of Electronic, Electrical and Communication Engineering, University of Chinese Academy of Sciences, Beijing 100049, China; 4 The College of Materials Science and Opto-Electronic Technology, University of Chinese Academy of Sciences, Beijing 100049, China

**Keywords:** MEMS biomimetic microphone, directional response, modal superposition, theoretical modeling, sound source localization

## Abstract

MEMS microphones inspired by the coupled ears of the fly *Ormia ochracea* have been extensively investigated for miniature, high-accuracy, and low-noise-floor sound source localization (SSL). However, most studies focus on the rocking-mode-dominated bidirectional polar response for SSL while neglecting the omnidirectional response of the bending mode, leaving other directional responses arising from the dual-mode superposition largely unexplored. Therefore, in this paper, based on a low-damping optical beam deflection (OBD) biomimetic microphone with a pronounced bending-mode omnidirectional response, various directional responses arising from the dual-mode superposition are identified and characterized. Both simulation and experimental results demonstrate that, under the dual-mode superposition, the directional responses of the OBD biomimetic microphone transition from the bidirectional polar pattern with asymmetric lobes to the two gradually overlapping circular patterns and eventually to the two nearly completely overlapping circular patterns, and this process is well described by the developed theoretical model. Moreover, we explore the SSL performance of the modal superposition-induced directional responses and demonstrate for the first time that the non-overlapping circular patterns have the same sinusoidal SSL potential as the bidirectional polar responses. This paper advances the understanding of modal superposition-induced directional responses and expands the variety of directional responses available for SSL in biomimetic microphones.

## 1. Introduction

To date, sound remains the most effective medium for human communication, and is expected to play an increasingly important role in efficient human–machine interaction [1] and environmental recognition [2] in the era of artificial intelligence. Among the information carried by sound, in addition to basic semantic information, directional cues constitute another critical aspect [3,4]. For example, the directional information allows robots to localize users, thereby enabling faster and more precise responses. Therefore, sound source localization (SSL) techniques have been developed to obtain the directional information and enhance our understanding of sound information. This technology has been widely applied across various fields, such as human–machine interaction [5], medical diagnostics [6], industrial production [7], security monitoring [8], and speech enhancement [9,10], etc. However, traditional microphone-array–based SSL technologies face practical challenges in compactness and system integration, mainly due to the fundamental size constraint [11]. Under this constraint, reducing the microphone spacing leads to increasing degradation of the directional cues captured by the array, including interaural phase difference (IPD) and interaural intensity difference (IID). Therefore, the microphone spacing is fundamentally constrained in microphone-array–based SSL, resulting in cumbersome and bulky systems with poor portability and limited integrability.

In order to overcome the size constraint and achieve miniaturization of SSL, MEMS microphones inspired by the coupled ears of the fly *Ormia ochracea* have been extensively studied due to their ability to amplify minute directional cues [12,13,14]. As yet, the biomimetic MEMS microphones have been developed with various diaphragm geometries (e.g., rectangular [15,16], square [17,18], and circular [19,20]) and demodulation systems (e.g., grating-based [15], capacitive [21], fiber-optic [22,23], piezoelectric [17], and optical beam deflection (OBD) [24]) to achieve high-performance SSL within a compact MEMS device. As a result, compared with the conventional microphone-array-based SSL technologies, the MEMS biomimetic microphones exhibit several notable advantages, including compact size, light weight, high computational efficiency, low noise, and a high signal-to-noise ratio [25].

For such MEMS biomimetic microphones, the directional response is the key factor in achieving SSL. Since the existing biomimetic microphones mimic the mechanically coupled ears of the fly *Ormia ochracea*, they exhibit two fundamental vibration modes: the rocking mode and the bending mode. Among these modes, the rocking mode responds to the sound pressure gradient [15], resulting in a bidirectional polar pattern that provides the primary directional cue for miniaturized SSL in biomimetic microphones [14]. Miles et al. first developed this type of MEMS biomimetic microphone [26], and the bidirectional polar pattern was initially considered to occur only at the rocking-mode resonant frequency [15]. This view influenced many subsequent studies, leading many researchers to focus only on the directional response at the rocking-mode resonant frequency [15,18,27]. Furthermore, some studies have observed the bidirectional polar patterns around the rocking-mode resonant frequency, which is beneficial for expanding the working frequency range of the biomimetic microphones [16]. Our previous study suggests that this phenomenon arises from the suppression of the omnidirectional component of the bending mode, leaving the directional response around the rocking-mode resonant frequency governed solely by the bidirectional polar pattern [24]. Based on this type of directional response with unique directionality, the biomimetic microphones can be arranged into arrays to achieve omnidirectional and multidimensional SSL [18,27,28].

However, in current studies, the bidirectional polar pattern has long been regarded as the only directional response available for SSL in the biomimetic microphones, while the omnidirectional response arising from the bending mode is typically neglected due to its lack of directionality [14]. This neglect has led to insufficient exploration of directional responses arising from the superposition of the rocking and bending modes, which fundamentally governs the directional behavior of the biomimetic microphones. Therefore, whether the dual-mode superposition can produce novel directional responses with the same SSL capability as the bidirectional polar pattern remains an open and interesting question. Such research can not only deepen our understanding of the modal superposition mechanisms in biomimetic microphones but also extend their capabilities and applications across diverse acoustic scenarios.

Therefore, in this paper, we investigate various directional responses arising from dual-mode superposition based on an OBD biomimetic microphone. The OBD biomimetic microphone employs a low-damping packaging design with a large enclosed back cavity, which results in a pronounced bending-mode omnidirectional response, thereby highlighting the important role of the omnidirectional response in the modal superposition process. Based on this biomimetic microphone, we identify and characterize various directional responses arising from the dual-mode superposition through simulations and experiments, and further explore the SSL potential of each directional response. In addition, we develop a theoretical model that enables a more precise quantitative description of the directional responses induced by the dual-mode superposition. This study demonstrates the possible directional responses of the biomimetic microphone arising from the dual-mode superposition and expands the types of directional responses applicable to SSL, thereby promoting further advances in biomimetic SSL research. In the following sections, the design, simulations, theoretical modeling, experimental validation, and related discussions of the OBD biomimetic microphone are described in detail.

## 2. Materials, Modeling, and Methods

### 2.1. Design of the OBD MEMS Biomimetic Microphone

**Figure 1:**
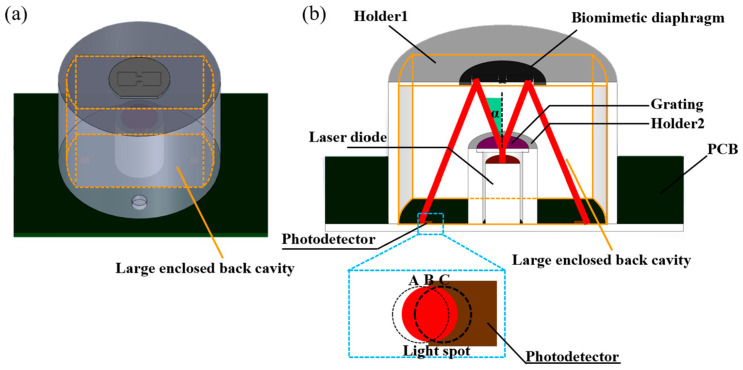
(**a**) Perspective and (**b**) section views of the OBD biomimetic microphone, highlighting the large enclosed back cavity. The bottom inset in (**b**) shows the states of the light spot on the photodetector when the biomimetic diaphragm vibrates.

Figure 1a,b are the perspective and section views of the OBD biomimetic microphone, respectively. The OBD biomimetic microphone consists of a biomimetic diaphragm supported by a housing (Holder 1) and an internal OBD demodulation system. The biomimetic diaphragm was fabricated on a Silicon-On-Insulator (SOI, Shin-Etsu, Tokyo, Japan) wafer via standard MEMS processes, including photolithography for pattern definition, deep reactive ion etching (DRIE) for high-aspect-ratio silicon etching, and reactive ion etching (RIE) for diaphragm release [23]. The fabricated biomimetic diaphragm (10 μm thick) features two square wings (3 mm × 3 mm) on either side, which are connected by a coupling bridge (1 mm × 1 mm). In addition, the coupling bridge with two torsional beams (0.1 mm × 0.2 mm) anchors the entire diaphragm to the substrate. In this configuration, the biomimetic diaphragm exhibits two types of vibration modes: the rocking mode, featuring out-of-phase vibrations, and the bending mode, featuring in-phase vibrations.

After the MEMS processing, the MEMS biomimetic diaphragm was integrated with an OBD demodulation system [24], as shown in Figure 1b. This demodulation system consists of a laser diode (DS-403R, Desheng Laser Co., Ltd., Shenzhen, Guangdong, China) vertically soldered to a PCB (JLCPCB, Shenzhen, Guangdong, China), a grating held by Holder 2 above the laser diode, two photodetectors (SMDB1.5, Beijing MinGuang Technology Co., Ltd., Beijing, China) positioned on either side of the laser, as well as the biomimetic diaphragm held by Holder1. The laser is a single-mode source equipped with a collimating lens and emits a 650-nm beam with a 1-mm diameter. The grating was fabricated by depositing a chromium layer on a glass substrate, followed by photolithography and etching. Given the grating period of 2.23 μm, the ±1st-order diffraction angle α in Figure 1b was calculated to be ±16.95° [24]. At such diffraction angles, the grating diffracted the laser beam into two separate beams, which were directed to either side of the diaphragm and reflected by its rear surface. To achieve this optical path, the heights of Holder1 and Holder2 were set to 21.57 mm and 11.61 mm, respectively. In addition, the photodetector has a 1.5-mm-square photosensitive area, and the distance between the photodetector and the laser was set to 9.57 mm, ensuring that the light spot initially corresponds to state B, as illustrated in the bottom inset of Figure 1b. Thus, as the diaphragm vibrates, the light spot on the photodetector moves back and forth between states A and C. This motion causes the photodetector’s output voltage to vary with the illuminated area of the light spot, enabling the demodulation of the biomimetic diaphragm’s vibrations. Moreover, sealing the bottom of Holder 1 with the PCB forms a relatively large enclosed back cavity, which includes both the diaphragm back cavity and the internal space of Holder 1, as shown in Figure 1.

### 2.2. Directional Response Simulation and SSL Potential Exploration of the OBD Biomimetic Microphone

**Figure 2:**
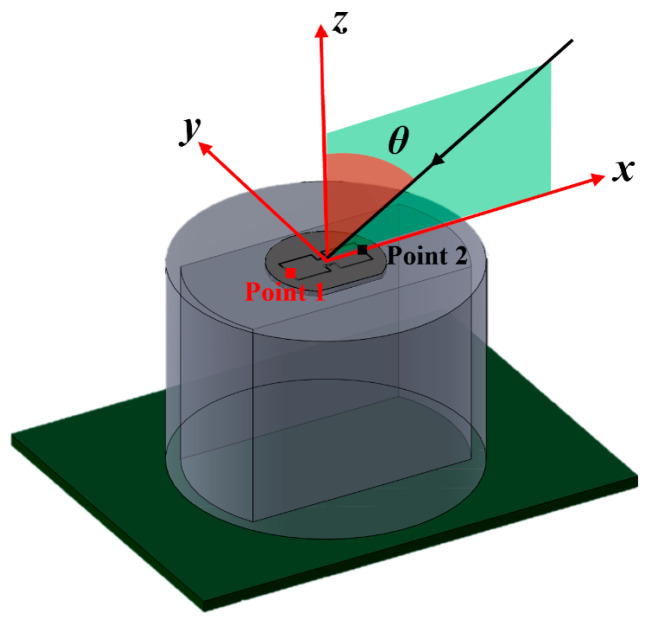
Simulation model of the OBD biomimetic microphone.

As an initial step in this study, the directional responses of the OBD biomimetic microphone were simulated (COMSOL Multiphysics 6.1) using the simulation model depicted in Figure 2. The simulation model includes the biomimetic diaphragm, Holder1, and the PCB that serves as a sealing component, but excludes other components of the OBD demodulation system due to their minimal contribution to the system’s acoustic behavior. When simulated using finite-element software, the simulation model was surrounded by a spherical air domain. Then, the air domain was divided into two layers. The inner concentric spherical region was assigned the “Thermoviscous Acoustics” interface to account for viscous and thermal losses associated with diaphragm vibration and sound propagation. The outer spherical shell was configured as the “Pressure Acoustics” interface to simulate the acoustic pressure excitation. In addition, except for the diaphragm, all other parts of the simulation model were fixed, and the “Solid Mechanics” interface was used to simulate the vibration responses of the biomimetic diaphragm. Based on this simulation setup, a plane sound wave propagating in the xz direction (1 Pa) was applied to the simulation domain at various incident angles (θ). Then, the directional responses were obtained by calculating the vibration amplitudes of the two distal-edge points of the diaphragm (Point 1 and Point 2, as shown in Figure 2).

**Figure 3:**
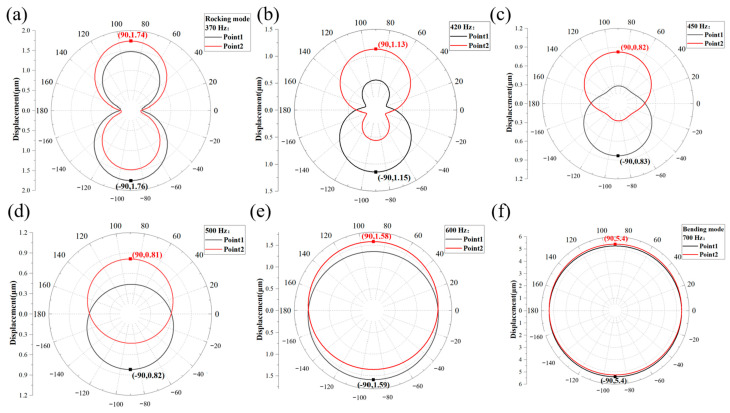
Simulated directional responses of the OBD biomimetic microphone from the rocking-mode resonant frequency to the bending-mode resonant frequency: (**a**) 370 Hz (rocking-mode resonant frequency); (**b**) 420 Hz; (**c**) 450 Hz; (**d**) 500 Hz; (**e**) 600 Hz; (**f**) 700 Hz (bending-mode resonant frequency).

Figure 3 presents the simulation results of the directional responses of the OBD biomimetic microphone. The simulated responses cover the frequency range from the rocking-mode resonant frequency at 370 Hz to the bending-mode resonant frequency at 700 Hz. These resonant frequencies were obtained from the frequency responses simulated under air damping conditions. In the simulation results, the directional responses exhibit two bidirectional polar patterns with asymmetric lobes at the rocking-mode resonant frequency, in which Point 1 shows the maximum directional response at −90° and Point 2 reaches the maximum directional response at 90°, as shown in Figure 3a. Then, as the incident frequency increases, the smaller sidelobes begin to shrink (Figure 3b) and gradually evolve into vertically distributed circular patterns (Figure 3c,d). Finally, the two circular patterns gradually overlap (Figure 3e), and at the bending-mode resonant frequency, the directional responses of Point 1 and Point 2 become two overlapping circular patterns, as illustrated in Figure 3f. Moreover, it is worth noting that the maximum values of these directional responses first decrease and then increase from the rocking-mode to the bending-mode resonant frequencies, which is consistent with the expected variation trend of the directional responses between the two resonant frequencies.

Subsequently, we explored the SSL potential of the simulated directional responses. According to the characteristics of the directional responses, two SSL strategies were adopted. First, for the directional responses of the bidirectional polar patterns with asymmetric lobes (Figure 3a,b), we employed a conventional strategy to calculate the combined directional response (CDR) [24]:

(1) $$CDR_{1}=\frac{Res1-Res2}{abs(Res1-Res2)}\times max(Res1,Res2)$$

where Res1 and Res2 are the directional responses of Point 1 and Point 2. In Equation (1), the multiplicand distinguishes the relative vibration amplitudes of Point 1 and Point 2 to determine the incident sector of the sound source (−180–0° or 0–180°), while the multiplier is used to construct the CDR as a single-valued function. In addition, for other directional responses resembling two gradually overlapping circular patterns, we adopted a strategy of directly subtracting the directional responses at the two points to construct the CDR:

(2) $$CDR_{2}=Res1-Res2$$

**Figure 4:**
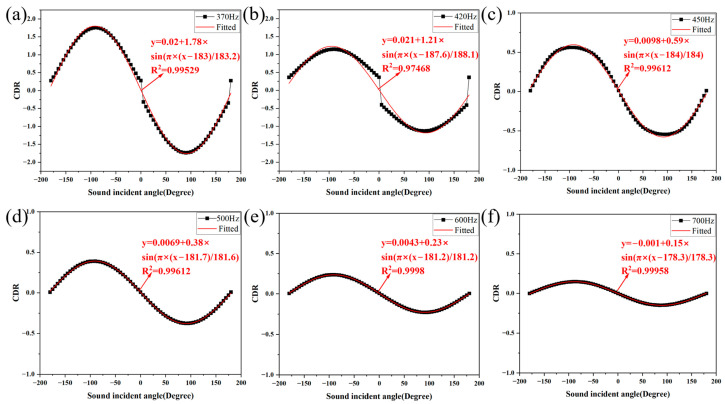
Calculated and fitted CDRs of the OBD biomimetic microphone from the rocking-mode resonant frequency to the bending-mode resonant frequency: (**a**) 370 Hz (rocking-mode resonant frequency); (**b**) 420 Hz; (**c**) 450 Hz; (**d**) 500 Hz; (**e**) 600 Hz; (**f**) 700 Hz (bending-mode resonant frequency). (**a**,**b**) are calculated by Equation (1) and others are calculated by Equation (2).

The calculated CDRs at the simulated frequencies are shown in Figure 4:

Figure 4a,b are the calculated CDRs of the bidirectional polar patterns with asymmetric lobes, calculated from Equation (1). It can be seen that these CDRs are well approximated by a sinusoidal function, indicating the sinusoidal SSL capability consistent with conventional understanding [24]. In addition, these CDRs exhibit discontinuities at 0° and 180°, and the prominence of these discontinuities increases with the growing asymmetry of the bidirectional polar patterns. Furthermore, Figure 4c–f show the CDRs of the directional responses resembling two gradually overlapping circular patterns, based on Equation (2). Compared with the CDRs of the bidirectional polar patterns with asymmetric lobes, these CDRs have no discontinuities and can be almost perfectly fitted by a sinusoidal function. Therefore, the two gradually overlapping circular patterns induced by modal superposition possess SSL capabilities similar to those of the bidirectional polar patterns. This behavior has not been reported in previous studies. Additionally, since the SSL capability of the two gradually overlapping circular patterns originates from the directional response difference between Points 1 and 2, the directional cues provided by the calculated CDRs (i.e., the amplitudes of the sinusoidally fitted functions) gradually weaken as the circular patterns continue to overlap. Therefore, when the overlap between the circular patterns becomes too high, it may ultimately limit their applicability for SSL.

### 2.3. Theoretical Modeling of the Directional Responses of the OBD Biomimetic Microphone

Subsequently, we developed a theoretical model of the directional responses of the OBD biomimetic microphone by solving the modal responses and establishing the modal superposition expressions.

Firstly, for the rocking mode driven by the gradient of the sound pressure, the modal response of the biomimetic microphone can be solved by the following differential equation [15]:

(3) $$\theta^{\prime \prime }(t)+2\omega_{1}\xi_{1}\theta^{\prime }(t)+{\omega_{1}}^{2}\theta (t)=M(t)/I$$

where θ(t) is the angular displacement of the biomimetic diaphragm, $\omega_{1}$ is the rocking-mode resonant frequency, $\xi_{1}$ is the corresponding damping ratio, and M(t) and I denote the moment applied by the sound pressure and the mass moment of inertia, respectively. Based on our previous research result [23], the amplitude $A_{1}$ and phase $\varphi_{1}$ of the steady-state solution to the above differential equation are:

(4) $$A_{1}=\frac{\left(\omega /2+L)\times (I_{A}/I)\times k\times P_{0}\times \sin(\varphi \right)}{-\omega^{2}\sin(\varphi_{1})+{\omega_{1}}^{2}\sin(\varphi_{1})-2\omega_{1}\xi_{1}\omega \cos(\varphi_{1})}$$

(5) $$\varphi_{1}=\arctan(\frac{\omega^{2}-{\omega_{1}}^{2}}{2\omega_{1}\xi_{1}\omega })$$

where w is the dimension of the coupling bridge, L is the dimension of the square wings, $I_{A}$ is the area moment of inertia of the rotation, k is the wave number, $P_{0}$ is the incident sound pressure, φ is the angle of incidence of the sound, and ω is the angular frequency of the incident sound. Furthermore, according to the expression of $\varphi_{1}$ and trigonometric identities, $\sin(\varphi_{1})$ and $\cos(\varphi_{1})$ can be expressed as:

(6) $$\sin(\varphi_{1})=\frac{\tan(\varphi_{1})}{\sqrt{1+\tan^{2}(\varphi_{1})}}$$

(7) $$\cos(\varphi_{1})=\frac{1}{\sqrt{1+\tan^{2}(\varphi_{1})}}$$

By substituting Equations (6) and (7) into Equation (4), we can finally obtain a phase-independent expression for $A_{1}$:

(8) $$A_{1}=\frac{\left(\omega /2+L)\times (I_{A}/I)\times k\times P_{0}\times \sin(\varphi \right)}{\sqrt{{\left(-\omega^{2}+{\omega_{1}}^{2}\right)}^{2}+{\left(2\omega_{1}\xi_{1}\omega \right)}^{2}}}$$

Secondly, for the bending mode driven by the average sound pressure, the modal response of the biomimetic microphone can be solved by the following differential equation [15]:

(9) $${A_{2}}^{\prime \prime }(t)+2\omega_{2}\xi_{2}{A_{2}}^{\prime }(t)+{\omega_{2}}^{2}A_{2}(t)=p(0,t)\times A/m$$

where $A_{2}$ is the translational displacement of the diaphragm, $\omega_{2}$ is the bending-mode resonant frequency, $\xi_{2}$ is the damping ratio, p(0,t) is the average sound pressure on the surface, A is the equivalent area of the diaphragm, and m is the effective mass. As described in our previous work [23], the amplitude $A_{2}$ and phase $\varphi_{2}$ of the steady-state solution of the above differential equation can be expressed as:

(10) $$A_{2}=\frac{P_{0}\times A/m}{-\omega^{2}\cos(\varphi_{2})+{\omega_{2}}^{2}\cos(\varphi_{2})+2\omega_{2}\xi_{2}\omega \sin(\varphi_{2})}$$

(11) $$\varphi_{2}=\arctan(\frac{2\omega_{2}\xi_{2}\omega }{{\omega_{2}}^{2}-\omega^{2}})$$

Furthermore, a phase-independent expression for $A_{2}$ can be obtained using the similar trigonometric identities as Equations (6) and (7):

(12) $$A_{2}=\frac{P_{0}\times A/m}{{\left(-\omega^{2}+{\omega_{2}}^{2}\right)}^{2}+{\left(2\omega_{2}\xi_{2}\omega \right)}^{2}}$$

Finally, based on the modal responses obtained above, the responses of the two square wings of the OBD biomimetic microphone can be calculated using the modal superposition method:

(13) $$A(Wing-1)=|A_{2}-A_{1}|+|A_{2}|$$

(14) $$A(Wing-2)=|A_{2}+A_{1}|+|A_{2}|$$

The above modal superposition expressions consist of two terms. In the first term, the rocking and bending modal responses are superposed in-phase or out-of-phase, which is a consensus that is widely accepted and documented in this field [26]. Moreover, the theoretical model proposed in this study innovatively introduces an omnidirectional baseline as the second term, which originates from the interaction between the in-phase vibration of the two square wings and the enclosed back cavity during the bending mode. This effect, which resembles a piston compressing and releasing air, is prominent in the bending mode but becomes almost negligible in the rocking mode due to the small cavity pressure variations caused by the out-of-phase vibrations of the two square wings.

**Figure 5:**
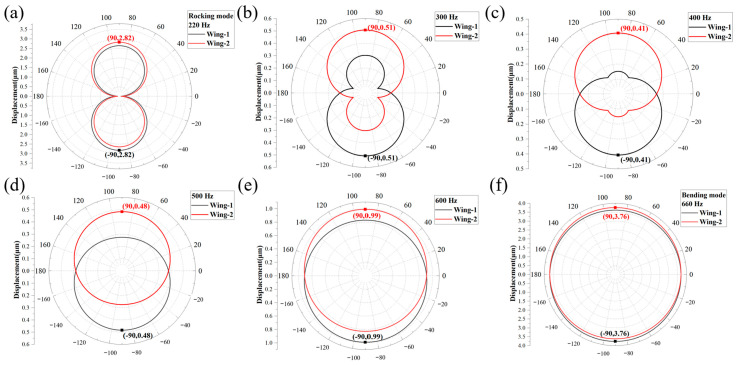
Theoretical calculation results of the directional responses of the OBD biomimetic microphone from the rocking-mode resonant frequency to the bending-mode resonant frequency: (**a**) 220 Hz (rocking-mode resonant frequency); (**b**) 300 Hz; (**c**) 400 Hz; (**d**) 500 Hz; (**e**) 600 Hz; (**f**) 660 Hz (bending-mode resonant frequency).

The specific parameters of the OBD biomimetic microphone are listed in Table 1, including the measured resonant frequencies and damping ratios (Section 3.1). Then, these parameters were substituted into the proposed theoretical model to calculate the directional responses at different frequencies:

From the theoretical calculation results in Figure 5, the patterns and variations of the directional responses are largely consistent with the simulation results—at the rocking-mode resonant frequency (Figure 5a), the OBD biomimetic microphone exhibits two bidirectional polar patterns with asymmetric lobes; then, as the frequency increases, the smaller sidelobes gradually shrink (Figure 5b) and the directional responses gradually become two non-overlapping circular patterns (Figure 5c,d); finally, the two circular patterns gradually overlap (Figure 5e) until they completely overlap at bending-mode resonant frequency (Figure 5f).

Nevertheless, there remain certain differences between the theoretical calculations and the simulation results. Firstly, the resonant frequencies employed in the simulations and those used in the theoretical calculation are different, leading to different frequencies being used to obtain the corresponding directional responses. Secondly, although the response magnitudes from the simulation and theoretical results are of the same order of magnitude, there are certain deviations between their exact values. For example, in the simulation results, the ratio of the maximum response magnitudes at the rocking-mode and bending-mode resonant frequencies is 1.74 µm to 5.4 µm, while this ratio is 2.82 µm:3.76 µm in the theoretical results. This discrepancy can be attributed to differences between the parameters used in the theoretical model and those adopted in the simulations of the OBD biomimetic microphone. Despite these discrepancies, the theoretical model proposed in this study can still accurately reproduce the superposition process of the directional responses of the biomimetic microphone, including the variations of the smaller sidelobes in the bidirectional polar patterns (e.g., at 300 and 400 Hz).

### 2.4. Experimental Methods

**Figure 6:**
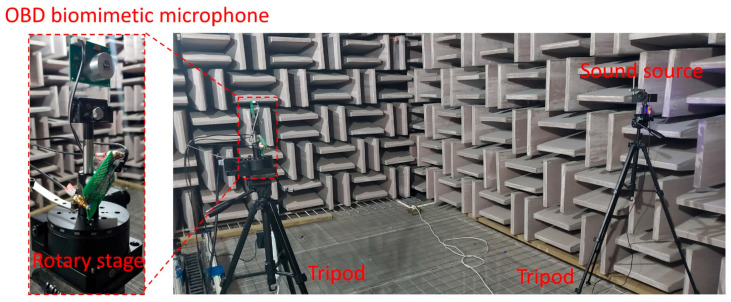
Experimental setup for measuring the directional responses of the OBD biomimetic microphone in an anechoic chamber.

Figure 6 shows the experimental setup for measuring the directional responses of the OBD biomimetic microphone in an anechoic chamber (background noise maintained at approximately 10–15 dBA). The anechoic chamber simulates an “infinite free sound field” by eliminating sound reflections and background noise, thereby enabling precise acoustic measurements. During the measurements, the OBD biomimetic microphone and sound source (FRS 5 X-8 Ohm, Visaton, Haan, Germany) were mounted face-to-face on two separate tripods at a fixed distance of 2 m. On one of the tripods, the OBD biomimetic microphone was installed on a rotating stage, allowing it to be rotated to any desired angle relative to the fixed sound source on the opposite tripod. During the measurement of directional responses, the sound source first emitted tones at the preset frequencies, while the rotation angle of the OBD biomimetic microphone was automatically controlled by a LabVIEW program and increased gradually to scan through the angles. Subsequently, the response signals corresponding to the two square wings of the OBD biomimetic microphone (labeled as CH1 and CH2) were acquired by a data acquisition card (National Instrument, USB-4431, Austin, TX, USA), and their response amplitudes were obtained through fast Fourier transform processing in LabVIEW (National Instruments, Austin, TX, USA). Finally, the directional responses were determined from the microphone’s response amplitudes at different incident angles, with a 10° step size.

## 3. Results

### 3.1. Measurement Result of the Frequency Responses of the OBD Biomimetic Microphone

**Figure 7:**
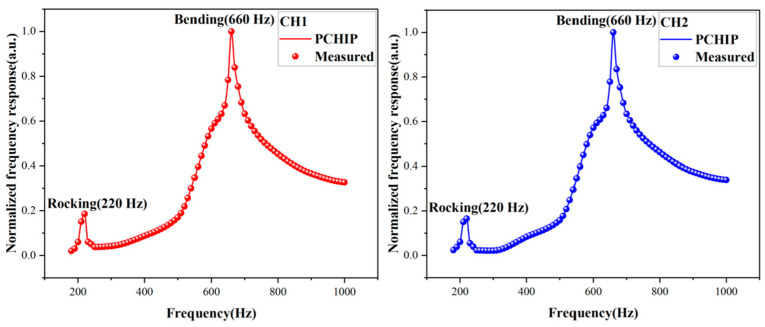
Normalized measured and interpolated frequency response curves of CH1 and CH2 of the OBD biomimetic microphone. The interpolation method used is the piecewise cubic Hermite interpolating polynomial (PCHIP).

Figure 7 illustrates the normalized measured frequency response curves of the OBD biomimetic microphone, conducted over the frequency range of 180 to 1000 Hz with a step size of 10 Hz. From the measurement results, two resonant frequencies can be obtained at 220 Hz and 660 Hz, corresponding to the rocking-mode and bending-mode resonant frequencies of the OBD biomimetic microphone, respectively. Subsequently, the measured frequency response curves were interpolated using the piecewise cubic Hermite interpolating polynomial (PCHIP) method to obtain smoothed curves with a resolution of 1 Hz, enabling accurate estimation of the damping ratios. The PCHIP method can generate smooth curves that pass through all data points, preserve the shape and monotonicity of the original data, and avoid overshoot or oscillations. Based on this method, the damping ratios can be obtained by the following formula [29]:

(15) $$\xi =\frac{\Delta f}{2f_{r}}$$

where Δf is the full width at half maximum, which can be determined by the spacing of two frequencies at 70.7% amplitude of the frequency response curve, and $f_{r}$ is the resonant frequency. As a result, the damping ratios can be calculated as $\xi_{1}=0.03637$ at the rocking-mode resonant frequency and $\xi_{2}=0.02197$ at the bending-mode resonant frequency, respectively.

### 3.2. Measurement Results of the Directional Responses of the OBD Biomimetic Microphone

**Figure 8:**
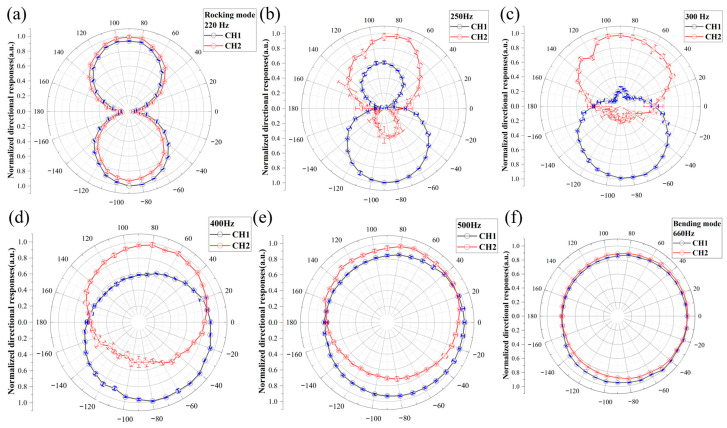
Measurement results of the directional responses of the OBD biomimetic microphone from the rocking-mode resonant frequency to the bending-mode resonant frequency: (**a**) 220 Hz (rocking-mode resonant frequency); (**b**) 250 Hz; (**c**) 300 Hz; (**d**) 400 Hz; (**e**) 500 Hz; (**f**) 660 Hz (bending-mode resonant frequency). Data points and error bars represent the mean values and standard deviations, respectively, calculated from five independent measurements.

Figure 8 illustrates the measurement results of the directional responses of the OBD biomimetic microphone. In the figures, the data points and error bars represent the mean values and standard deviation of five independent measurements. As shown in Figure 8a, the directional responses present two asymmetric bidirectional polar patterns at the rocking-mode resonant frequency of 220 Hz. Then, as the frequency increases to 250 Hz, the smaller sidelobes gradually shrink (Figure 8b). Subsequently, at 300 Hz, the smaller sidelobes are significantly suppressed but remain slightly visible, as shown in Figure 8c, and by 400 Hz, the measured directional responses gradually evolve into two non-overlapping circular patterns (Figure 8d). Finally, with increasing frequency up to 500 Hz, the two circular patterns gradually overlap (Figure 8e), and the circular patterns closely overlap at the bending-mode resonant frequency of 660 Hz, as shown in Figure 8f. Compared with the simulation and theoretical results, the measured directional response patterns and their frequency-dependent behavior are generally consistent, although some measurement errors and discrepancies still remain and warrant further analysis. Firstly, at 220, 400, 500, and 660 Hz, where the OBD biomimetic microphone exhibits relatively high frequency responses (as shown in Figure 7), the measured directional responses show minimal uncertainty, as indicated by the small error bars. As a result, the measured patterns at these frequencies are in good agreement with both the simulation predictions and theoretical results. Secondly, at the frequencies with lower frequency responses (250 Hz and 300 Hz), CH1 exhibits relatively low uncertainty in the measured directional responses, as indicated by the small error bars, whereas CH2 shows higher uncertainty with larger error bars. Therefore, the directional responses of CH1 more closely match the simulation and theoretical results, whereas CH2 exhibits a certain degree of distortion in comparison. This distortion is caused by both the reduced demodulation sensitivity of CH2 and the diminished mechanical sensitivity of the OBD biomimetic microphone. Specifically, the decreased demodulation sensitivity of CH2 is attributed to its initial operating point slightly deviating from the quadrature operating point [24], whereas the diminished mechanical sensitivity of the OBD biomimetic microphone is due to its smaller frequency response at the two non-resonant frequencies, as illustrated in Figure 7. Therefore, under constraints in demodulation and response sensitivity, CH2 exhibits larger measurement errors in the directional responses, particularly in the low-response angular range from 0° to −180°, resulting in distortion of the measured directional patterns.

**Figure 9:**
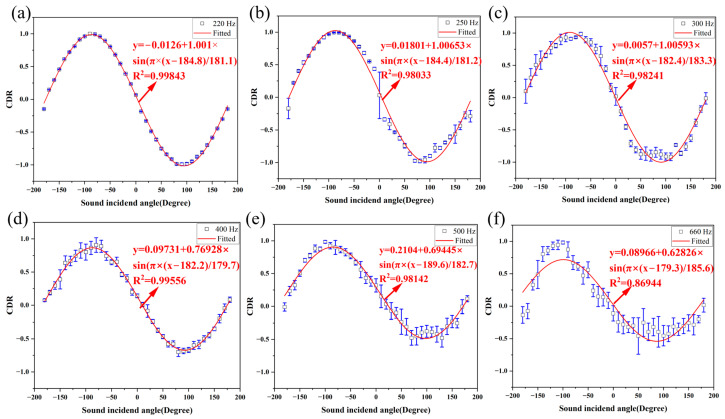
Calculated and fitted CDRs of the OBD biomimetic microphone from the rocking-mode resonant frequency to the bending-mode resonant frequency: (**a**) 220 Hz (rocking-mode resonant frequency); (**b**) 250 Hz; (**c**) 300 Hz; (**d**) 400 Hz; (**e**) 500 Hz; (**f**) 660 Hz (bending-mode resonant frequency). (**a**,**b**) are calculated by Equation (1) and the others are calculated by Equation (2). Data points and error bars represent the mean values and standard deviations, respectively, calculated from five independent measurements.

Subsequently, we evaluated the SSL potential of the measured directional responses by calculating the CDRs at the different frequencies, as shown in Figure 9. First, the CDRs of the asymmetric bidirectional polar patterns at 220 Hz and 250 Hz were calculated using Equation (1), as shown in Figure 9a,b, respectively. It can be seen that the CDR at 220 Hz can be well fitted by a sinusoidal function, with coefficients of determination (R^2^) of 0.99843 and minimal error bars. This suggests that the OBD biomimetic microphone exhibits a high-precision sinusoidal SSL capability at the rocking-mode resonant frequency. Furthermore, for the bidirectional polar patterns with reduced sidelobes, the CDR at 250 Hz can still be fitted with a sinusoidal function, despite the slightly lower fitting quality (R^2^ = 0.98033). Moreover, the CDR exhibits relatively large errors at 0° and ±180°, which correspond to the discontinuities in the simulated CDR (Figure 4b). This phenomenon occurs because the responses of CH1 and CH2 are very close in magnitude at these angles, where the measurement errors cause a reversal in their relative amplitudes, leading to sign reversals in the CDR calculated using Equation (1). Secondly, the CDRs of the directional responses resembling two gradually overlapping circular patterns were calculated using Equation (2), as shown in Figure 9c–f. In Figure 9c,d,f, the calculated CDRs exhibit no discontinuities and can be well fitted by a sinusoidal function, which is consistent with the simulation results. Specifically, the R^2^ values of the CDRs at 300 Hz and 400 Hz are 0.99556 and 0.98241, respectively, and it can be observed that the larger CDR errors are concentrated in the angular range from −180° to 0°. According to the measured directional responses (Figure 8c,d), this phenomenon can be attributed to the larger measurement errors caused by the weak response of CH2 in this angular range. Furthermore, for the more overlapping circular patterns at 500 Hz, the CDR can also be fitted by a sinusoidal function (R^2^ =0.98142). However, this CDR exhibits relatively large measurement errors in the angular range from 0° to 180°. From the measured directional responses (Figure 8e), neither CH1 nor CH2 exhibits significant measurement errors in this angular range. Therefore, this phenomenon can be attributed to the increased overlap between the two directional responses over this range of incident angles, which weakens the directional cues and leads to increased measurement errors. Finally, at 660 Hz, where the two circular patterns exhibit excessive overlap, the calculated CDR shows the lowest agreement with a sinusoidal function (R^2^ = 0.8746) and the largest measurement errors, as shown in Figure 9f. This indicates that even when the measurement errors of the CH1 and CH2 are both low, the overlap of the circular patterns can still blur the directional cues, thereby significantly increasing the SSL errors of the OBD biomimetic microphone. Overall, these experimental results demonstrate that, except when the two circular patterns are excessively overlapped, the two circular patterns can exhibit the sinusoidal SSL potential similar to that of bidirectional polar patterns with asymmetric lobes.

## 4. Discussion

In this study, we investigate the directional responses of the OBD biomimetic microphone under the dual-mode superposition through a combination of simulations, theoretical modeling, and experimental validation. Compared with previous studies [14], this work reveals additional novel directional responses arising from the dual-mode superposition, and demonstrates that the directional responses of the two gradually overlapping circular patterns exhibit the same sinusoidal SSL potential as the bidirectional polar patterns with asymmetric lobes. Moreover, in contrast to previous studies that attributed the directional responses of biomimetic microphones solely to the superposition of the two modal responses [29], the theoretical model developed and validated in this work demonstrates that the inclusion of an omnidirectional baseline is essential for an accurate description of the directional responses under dual-mode superposition. Therefore, this study not only deepens the understanding of directional responses induced by the dual-mode superposition but also broadens the range of responses available for SSL in biomimetic microphones.

Secondly, based on the theoretical modeling proposed in this study, three key physical factors governing the directional responses of biomimetic microphones can be distilled, namely vibration modes, damping and modal superposition manners. These three physical factors influence the directional responses in distinct yet interrelated ways. The first physical factor, the vibration mode, is the decisive factor in the directional responses of biomimetic microphones. Under the enclosed back cavity, the rocking mode driven by sound pressure gradient gives rise to the bidirectional polar pattern, whereas the bending mode driven by average sound pressure results in the omnidirectional directional response. The second physical factor is the damping of each vibration mode, which constitutes the critical factor in the modal superposition equations (Equations (13) and (14)). For the OBD biomimetic microphone with a large enclosed back cavity in this study, the damping ratios are 0.03637 and 0.02197, respectively. By comparison, for the fiber-optic biomimetic microphone previously studied, which has a small enclosed back cavity, the damping ratios are 0.453 and 0.374, respectively [23]. Therefore, it can be concluded that the size of the enclosed back cavity in the biomimetic microphone affects the damping, with a larger enclosed back cavity resulting in lower damping. Furthermore, in the fiber-optic biomimetic microphone with a small enclosed back cavity, the omnidirectional response of the bending mode is significantly suppressed, and thus the response across the entire frequency range is dominated by the bidirectional polar patterns [22]. In this study, the OBD biomimetic microphone with a large enclosed back cavity has a relatively pronounced omnidirectional response of the bending mode (see Figure 3a,f), and thus the directional responses across the entire frequency range exhibit the results of the dual-mode superposition. Therefore, it can be concluded that the damping affects the responses of the two vibration modes and ultimately leads to different directional responses via modal superposition. The final physical factor is the modal superposition manner. Compared with the conventional understanding that the directional responses of biomimetic microphones arise directly from the in-phase or out-of-phase superposition of the two modal responses [27], the modal superposition manner proposed in this study, which incorporates the omnidirectional baseline, can more accurately describe the modal superposition mechanism underlying the directional responses of the biomimetic microphone. Therefore, the modal superposition manner is the ultimate factor determining the directional responses of biomimetic microphones at different frequencies. Furthermore, based on the three key physical factors distilled above, three potential approaches for future control of the directional responses of biomimetic microphones can be inferred, including creating novel vibration modes, regulating the damping of the microphones, and leveraging the discovered modal superposition mechanism.

Thirdly, although this study successfully predicted and verified various directional responses of the OBD biomimetic microphone under the dual-mode superposition, there are discrepancies in the frequencies corresponding to the directional responses among the theoretical model, simulations, and experiments, as shown in Table 2. Here, taking the experimentally measured frequencies as the reference, the reasons for these frequency differences are discussed separately. First, the frequency discrepancy between the simulations and the experiments can be attributed to differences in the two resonant frequencies, which lead to modal superposition occurring at different frequency locations. Generally, the resonant frequency of the biomimetic diaphragm can be expressed as:

(16) $$f=\frac{1}{2\pi }\sqrt{\frac{k_{eff}}{m_{eff}}}\sqrt{1-\xi^{2}}$$

where $k_{eff}$ and $m_{eff}$ denote the effective stiffness and effective mass, respectively, and ξ is denotes the damping ratio. Among these parameters, the effective mass is primarily determined by the diaphragm’s geometry, material properties, and boundary conditions, whereas the damping ratio is mainly determined by the sensor packaging and the air damping modeling in the finite-element software. Since the diaphragm geometry, boundary conditions, and packaging configuration of the biomimetic microphone have been carefully reproduced in the finite-element simulations, the effective mass and damping are unlikely to be the dominant sources of the observed frequency discrepancies. Therefore, the following discussion mainly focuses on the influence of effective stiffness on the resonant frequencies. In the simulations, the biomimetic diaphragm is assumed to be an idealized structure; therefore, the initial residual stress within the diaphragm is not considered. However, in practical MEMS fabrication, residual stress is inevitably introduced during the diaphragm release process. Since the biomimetic diaphragm is fabricated using an SOI-based MEMS process, tensile stress is typically induced in the device-layer silicon bonded to the buried oxide layer. Therefore, after the removal of the buried oxide layer, residual stress redistribution induces slight out-of-plane static deformation of the silicon diaphragm. This pre-deformed geometry reduces the effective geometric stiffness of the structure, thereby decreasing the resonant frequency compared with the ideal flat and perfectly clamped condition assumed in the simulations. Moreover, the rocking-mode resonant frequency is lower than that of the bending mode; therefore, the original effective stiffness of the rocking mode is lower than that of the bending mode. Consequently, for the same magnitude of effective stiffness reduction, the rocking mode exhibits a greater decrease in resonant frequency. This can also explain why the error in the rocking-mode resonant frequency is greater than that of the bending mode, as shown in Table 2. Furthermore, the frequency errors between the theoretical and experimental directional responses are relatively smaller compared to those from the simulation, as shown in Table 2. In the theoretical calculations, the modal responses of the rocking mode and bending mode exhibit different magnitudes at different frequencies. Therefore, in the final modal superposition formulation, the relative contributions of the two modal responses at different frequencies determine the directional response pattern at each frequency. As a result, the discrepancies can be attributed to errors in key parameter estimation, such as the mass moment of inertia I, equivalent area of the diaphragm A, and the effective mass m, which affect the modal responses and thus the directional response patterns after the modal superposition. Therefore, a more accurate estimation of these key parameters can further reduce the discrepancies between the theoretical model and the experimental results.

Finally, the limitations of the present study should be noted. Specifically, although the work investigated the directional responses of the biomimetic microphone under the dual-mode superposition and preliminarily explored its SSL potential, it did not involve practical SSL implementation or validation. In the future, the SSL performance of this OBD biomimetic microphone can be demonstrated through estimated angles, angular errors, ambiguity analysis, or robustness tests. However, prior to this, several potential improvements and key factors that warrant careful attention should be considered. First, since the non-overlapping circular patterns occur between the two resonant frequencies, the mechanical sensitivity of the biomimetic microphone is low. Therefore, to prevent distortion of the directional responses and reduce measurement errors of the CDR, both demodulation channels should maintain high sensitivity and signal-to-noise ratio. Second, the degree of non-overlap of the circular directional responses is a key factor to obtain the directional cues enabling SSL for the biomimetic microphone. Therefore, directional responses with excessively overlapping circular patterns should not be used for SSL, as this weakens directional cues and leads to larger CDR localization errors. Following these improvements and careful consideration of key factors, the OBD biomimetic microphone is expected to enable practical SSL through array-based integration.

## 5. Conclusions

In this study, we demonstrate the directional responses of the developed OBD biomimetic microphone under the dual-mode superposition. The simulations, theoretical modeling, and experimental validation consistently show that through the superposition of the rocking-mode bidirectional patterns with the bending-mode omnidirectional responses, the directional responses of the OBD biomimetic microphone transition from the bidirectional polar patterns with asymmetric lobes to the two gradually overlapping circular patterns and eventually to the two nearly completely overlapping circular patterns. Notably, the investigation into the SSL potential of the different directional responses reveals that the two gradually overlapping circular patterns can exhibit a sinusoidal SSL potential similar to the bidirectional polar responses. Furthermore, the developed and validated theoretical model suggests that incorporating the omnidirectional baseline into the final modal superposition is likely necessary to more accurately describe the directional response process of the biomimetic microphone. Therefore, these findings provide further insight into modal superposition-induced directional responses and extend the range of directional responses in biomimetic microphones that can be leveraged for SSL.

## Figures and Tables

**Table 1:** Parameters used in the theoretical calculation.

Parameter	Description	Formula	Value
L	dimension of the square wings	N/A	$3\times 10^{-3}\;m$
w	dimension of the coupling bridge	N/A	$1\times 10^{-3}\;m$
$I_{A}$	area moment of inertia of the rotation [23]	$\frac{2}{3}L^{4}+L^{3}w+\frac{1}{2}L^{2}w^{2}+\frac{1}{12}w^{4}$	$8.56\times 10^{-11}{\;m}^{4}$
a	torsional beam length	N/A	$2\times 10^{-4}\;m$
b	torsional beam width	N/A	$1\times 10^{-4}\;m$
J	torsional constant [23]	$0.321\times ab^{3}$	$3.12\times 10^{-20}{\;m}^{4}$
G	shear modulus [23]	N/A	$8.05\times 10^{10}\;\mathrm{Pa}$
$k_{t}$	torsional stiffness [23]	$\frac{1}{2}\times \frac{GJ}{a}$	$6.28\times 10^{-6}\;\mathrm{kg}\cdot {\;m}^{2}$/${\;s}^{2}$
$\omega_{1}$	angular frequency at the rocking-mode frequency	N/A	$1.38\times 10^{3}\;\mathrm{rad}/s$
*I*	mass moment of inertia [23]	$k_{t}/\omega_{1}^{2}$	$3.29\times 10^{-12}\;\mathrm{kg}\cdot {\;m}^{2}$
*k*	wave number at the rocking-mode frequency	N/A	$12.09{\;m}^{-1}$
$P_{0}$	sound pressure	N/A	1 Pa
$\xi_{1}$	damping ratio at the rocking-mode frequency	N/A	0.03637
φ	angle of incidence of the sound	N/A	−180 to 180 deg
A	equivalent area	N/A	$1.9\times 10^{-5}\;m$
μ	Poisson’s ratio	N/A	0.18
E	Young’s modulus [23]	N/A	$1.9\times 10^{11}\;\mathrm{Pa}$
*t*	thickness of the diaphragm	N/A	$1\times 10^{-5}\;m$
$k_{b}$	bending stiffness [23]	$\frac{Et^{3}}{{\left(w-b\right)}^{2}}$	$234.57\;\mathrm{kg}/{\;s}^{2}$
$\omega_{2}$	angular frequency at the bending-mode frequency	N/A	$4.15\times 10^{3}\;\mathrm{rad}/s$
m	effective mass [23]	$\frac{k_{b}}{\omega_{2}^{2}}$	$1.36\times 10^{-5}\;\mathrm{kg}$
$\xi_{2}$	damping ratio at the bending-mode frequency	N/A	0.02197

**Table 2:** Frequencies of the corresponding directional response patterns obtained from the simulation, theoretical and experimental results, and the relative error of the experimental results with respect to the simulation results ($RE_{E\_vs\_S}$), and the relative error of the experimental results with respect to the theoretical results ($RE_{E\_vs\_T}$).

	Rocking Mode					Bending Mode
Simulations	370 Hz	420 Hz	450 Hz	500 Hz	600 Hz	700 Hz
Theory	220 Hz	300 Hz	400 Hz	500 Hz	600 Hz	660 Hz
Experiment	220 Hz	250 Hz	300 Hz	400 Hz	500 Hz	660 Hz
$RE_{E\_vs\_S}$	40.5%	40.5%	33.3%	20%	16.7%	5.7%
$RE_{E\_vs\_T}$	0%	16.7%	25%	20%	16.7%	0%

## Data Availability

The data that support the findings of this study are available from the corresponding author upon reasonable request.

## References

[1] Zolfagharinejad M., Büchel J., Cassola L., Kinge S., Syed G.S., Sebastian A., van der Wiel W.G. (2025). Analogue Speech Recognition Based on Physical Computing. Nature.

[2] Xu H., Chen H., Liu X., Ren H. (2025). Improving One-Dimensional-Based Environmental Sound Classification Models with Progressive Projection Knowledge Distillation from Two-Dimensional-Based Models. Eng. Appl. Artif. Intell..

[3] Rascon C., Meza I. (2017). Localization of Sound Sources in Robotics: A Review. Robot. Auton. Syst..

[4] Jekaterynczuk G., Piotrowski Z. (2023). A Survey of Sound Source Localization and Detection Methods and Their Applications. Sensors.

[5] Jo H.-M., Kim T.-W., Kwak K.-C. (2025). Sound Source Localization Using Deep Learning for Human–Robot Interaction Under Intelligent Robot Environments. Electronics.

[6] Demené C., Robin J., Dizeux A., Heiles B., Pernot M., Tanter M., Perren F. (2021). Transcranial Ultrafast Ultrasound Localization Microscopy of Brain Vasculature in Patients. Nat. Biomed. Eng..

[7] Yu L., Gong Z., Chu N., Ning Y., Zheng Y., Hou P. (2021). Adaptive Imaging of Sound Source Based on Total Variation Prior and a Subspace Iteration Integrated Variational Bayesian Method. IEEE Trans. Instrum. Meas..

[8] Nguyen Q., Shen G., Choi J. (2016). Sound Detection and Localization in Windy Conditions for Intelligent Outdoor Security Cameras. Circuits Syst. Signal Process..

[9] Tao T., Zheng H., Yang J., Guo Z., Zhang Y., Ao J., Chen Y., Lin W., Tan X. (2022). Sound Localization and Speech Enhancement Algorithm Based on Dual-Microphone. Sensors.

[10] Xenaki A., Boldt J.B., Christensen M.G. (2018). Sound Source Localization and Speech Enhancement with Sparse Bayesian Learning Beamforming. J. Acoust. Soc. Am..

[11] Liu H., Currano L., Gee D., Helms T., Yu M. (2013). Understanding and Mimicking the Dual Optimality of the Fly Ear. Sci. Rep..

[12] Robert D., Read M.P., Hoy R.R. (1994). The Tympanal Hearing Organ of the Parasitoid Fly *Ormia ochracea* (*Diptera*, *Tachinidae*, *Ormiini*). Cell Tissue Res..

[13] Miles R.N., Robert D., Hoy R.R. (1995). Mechanically Coupled Ears for Directional Hearing in the Parasitoid Fly *Ormia ochracea*. J. Acoust. Soc. Am..

[14] Ishfaque A., Kim B. (2018). Fly *Ormia ochracea*-Inspired MEMS Directional Microphone: A Review. IEEE Sens. J..

[15] Miles R.N., Su Q., Cui W., Shetye M. (2009). A Low-Noise Differential Microphone Inspired by the Ears of the Parasitoid Fly *Ormia ochracea*. J. Acoust. Soc. Am..

[16] Miles R.N., Cui W., Su Q.T., Homentcovschi D. (2015). A MEMS Low-Noise Sound Pressure Gradient Microphone with Capacitive Sensing. J. Microelectromech. Syst..

[17] Kuntzman M.L., Hall N.A. (2014). Sound Source Localization Inspired by the Ears of the *Ormia ochracea*. Appl. Phys. Lett..

[18] Wilmott D., Alves F., Karunasiri G. (2016). Bio-Inspired Miniature Direction Finding Acoustic Sensor. Sci. Rep..

[19] Rahaman A., Kim B. (2021). Sound Source Localization in 2D Using a Pair of Bio–Inspired MEMS Directional Microphones. IEEE Sens. J..

[20] Mackie D.J., Jackson J.C., Brown J.G., Uttamchandani D., Windmill J.F.C. (2014). Directional Acoustic Response of a Silicon Disc-Based Microelectromechanical Systems Structure. IET Micro Nano Lett..

[21] Touse M., Sinibaldi J., Simsek K., Catterlin J., Harrison S., Karunasiri G. (2010). Fabrication of a Microelectromechanical Directional Sound Sensor with Electronic Readout Using Comb Fingers. Appl. Phys. Lett..

[22] Ren D., Liu X., Zhang M., Gao R., Qi Z.-M. (2021). Low-Frequency Bidirectional Microphone Based on a Combination of Bionic MEMS Diaphragm and Fiber Acousto-Optic Transducer. IEEE Sens. J..

[23] Ren D., Yang X., Qi Z. (2024). Fast Demodulated White-Light Interferometry-Based Measurement and Theoretical Verification of Mechanical Sensitivity of MEMS Biomimetic Microphone with Fiber-Optic Fabry–Pérot Interferometer. IEEE Sens. J..

[24] Ren D., Qi Z.-M. (2021). An Optical Beam Deflection Based MEMS Biomimetic Microphone for Wide-Range Sound Source Localization. J. Phys. D Appl. Phys..

[25] Rahaman A., Kim B. (2020). Sound Source Localization by *Ormia ochracea*-Inspired Low-Noise Piezoelectric MEMS Directional Microphone. Sci. Rep..

[26] Miles R.N., Hoy R.R. (2006). The Development of a Biologically-Inspired Directional Microphone for Hearing Aids. Audiol. Neurotol..

[27] Rahaman A., Kim B. (2022). An mm-Sized Biomimetic Directional Microphone Array for Sound Source Localization in Three Dimensions. Microsyst. Nanoeng..

[28] Waqar A., Khan A., Kim B., Park D. (2024). *Ormia ochracea* Inspired Single-Microphone Approach for 3-D Sound Localization. IEEE Sens. Lett..

[29] Liu C. (2012). Foundations of MEMS.

